# Slippery, Water‐Infused Membrane with Grooved Nanotrichomes for Lubricating‐Induced Oil Repellency

**DOI:** 10.1002/advs.202103950

**Published:** 2022-02-09

**Authors:** Young A Lee, Seohyun Cho, Seounkyun Choi, O‐Chang Kwon, Sun Mi Yoon, Seong Jin Kim, Kyoo‐Chul Park, Seok Chung, Myoung‐Woon Moon

**Affiliations:** ^1^ Extreme Materials Research Center Korea Institute of Science and Technology (KIST) Seoul 02792 Republic of Korea; ^2^ Department of Biomicrosystem Technology Korea University Seoul 02841 Republic of Korea; ^3^ School of Mechanical Engineering Korea University Seoul 02841 Republic of Korea; ^4^ Department of Mechanical Engineering Northwestern University Evanston IL 60208 USA; ^5^ KU‐KIST Graduate School of Converging Science and Technology Korea University Seoul 02841 Republic of Korea

**Keywords:** grooved nanotrichome, slippery water infused membrane, SWIS oil scooper, thick and stable water lubrication layer

## Abstract

Water, abundant and ubiquitous in nature, is an easy yet powerful resource for the creatures to survive by putting together with their topologies interfacing their living environment. Here, a slippery, water‐infusing surface (SWIS) that retains a thick and stable water layer on the membrane is presented, robustly maintaining the oil repellency against the pressure and friction of immiscible liquids. Inspired by the plant trichome structures and their function, grooved nanotrichome, formed on the fibrous membrane by the oxygen plasma etching, induces robust water lubrication on the SWIS. SWIS membrane repels and separates highly viscous and adhesive oils in air and underwater by preventing oils from adhering to the lubricating surface. Repeated tests both in air and underwater confirm the antiadhesion and self‐cleaning properties of the SWIS. The SWIS oil scooper, fixed on a frame with a handle, successfully collects spilled oil on a pilot‐scale oil spill site and a real ocean oil spill site by simply scooping and recovering the oil. In addition, SWIS membrane is expected to help protect environments with further applications such as oil‐wastewater treatment and oil separation in food.

## Graphical abstract

Assisted with water, a natural lubricant for many plants or insects, a cellulose membrane with grooved nanotrichomes is suggested for a slippery, water‐infused surface (SWIS). With the stable and thick water lubricating layer, the SWIS membrane recovers the spilled oil by scooping and collecting, which is easy to operate, inexpensive to manufacture, and environmental‐friendly solution to water pollution.

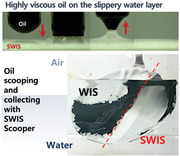

## 1 Introduction

Living creatures in nature have been evolved with distinct surface topographies at the micro‐ or nanoscale or their hierarchical hybrids as part of their survival strategies for persistent interactions with water.^[^ ^1^ ^]^ According to the degree of water wetting, the surfaces render water‐repellent or water‐attractive, which has been explored for functions of self‐cleaning,^[^ ^2^ ^]^ antiadhesion,^[^ ^3^ ^]^ water harvesting,^[^ ^4^ ^]^ and oil repellency^[^ ^5^ ^]^ on engineering structures, biological devices, and filtration membranes. Another category of functional wettability was defined by a structured surface infused with a lubricant liquid, referred to as a liquid‐infused surface (LIS). Previous works mainly presented an example of LIS inspired by the peristome of the *Nepenthes* pitcher plant, on which the microstructures sustained a thin lubricating layer.^[^ ^6^ ^]^ LISs have demonstrated extraordinary performance in antiadhesion, drag reduction, anti‐icing, and repellency against water.^[^ ^7^ ^]^ To establish a uniform surface lubrication, they choose hydrocarbon‐based liquids with lower surface energy, density and vapor pressure.^[^ ^8^ ^]^ The lubricant, however, was easily damaged by the impact and shear flow of the target liquids causing the performance degradation of LIS.^[^ ^9^ ^]^ Additionally, the harmful effect of fluoride‐containing oil lubricants seemed not to be verified.^[^ ^10^ ^]^ Water, an abundant natural resource with high surface energy (72 mN m^−1^) and density, would be an excellent lubricant candidate. Many works have proposed water‐infused surfaces (WIS) for repelling immiscible liquids by enhancing water‐surface interaction and showed remarkable separation efficiency in the filtration system.^[^ ^11^ ^]^ Still, the practical application of WIS has been restricted due to its uncertain durability, low drain flux, complicated fabrication, and difficult scale‐up. The porous cellulose membranes were widely used in WIS to separate immiscible liquids owing to its good water‐wetting behavior and high drainage capability.^[^ ^12^ ^]^ However, infused water mostly wicked into the porous body structure, depleting the water lubrication at the interface between the membrane and the target liquids (i.e., oil). The surface might result in oil fouling and lubricant loss. Therefore, designing a surface structure for firm and complete water lubrication at the air‐solid interface of the porous membrane would be a critical challenge to expand the usage of water as a lubricant.

**Figure 1:**
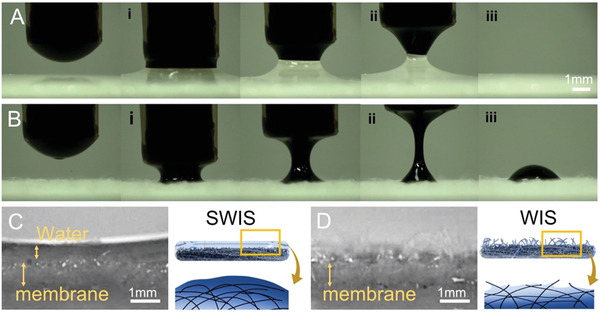
Oil repellency behavior by oil attachment and detachment procedure on the fibrous membranes with the SWIS and the WIS under the same condition. A heavy oil droplet was touched, spread, and lifted on the membranes with A) the SWIS and B) the WIS, which were covered C) with and D) without the thick water layer, respectively. Oil on (A) and (B) was LSFO (Low sulfur fuel oil).

In this work, we fabricated a cellulose membrane with grooved nanotrichomes (GNTs) for rendering a slippery, water‐infused surface (SWIS). The superhydrophilic GNTs artificially formed on a fibrous membrane through oxygen plasma etching were found to induce the stable and thick water lubricating layer. The water layer was steady enough to protect the surface from fouling by the pressure of immiscible liquids. When highly viscous oil was gently pressed against a membrane with SWIS, the oil droplet deformed mildly, and its bottom spread slightly all over the water‐covered surface (**Figure** **1**A,i). Upon lifting the oil droplet, an oil‐water bridge was formed due to the oil‐water surface tension. The oil‐water contact line receded as the oil slipped over water, occurring necking at the oil‐water interface of the bridge (Figure 1A,ii). Continuous lifting separated the oil droplet from the water layer, leaving no oil residue on the water‐air interface (Figure 1A,iii and Movie S1 right, Supporting Information). On the other hand, immediately after oil droplets touched under the same condition, the pristine membrane surface, defined as a WIS, was fouled (Figure 1B,i). The oil bridge was gradually necking and thinning with lifting (Figure 1B,ii) and was finally broken by pinch‐off. The oil left a significant residue on the WIS, indicating that the high interfacial adhesion between oil and the fibers appeared on the top region of the membrane (Figure 1B,iii and Movie S1 left, Supporting Information). It suggested that the SWIS fibers with GNTs provided a crucial matrix for water covering the entire surface (Figure 1C), while numerous fibers on the pristine membrane without GNTs stayed above the water layer, becoming the sites for water lubrication discrete and oil fouling (Figure 1D).

## 2 Results

### 2.1 SWIS Membrane with GNT Fibers

**Figure 2:**
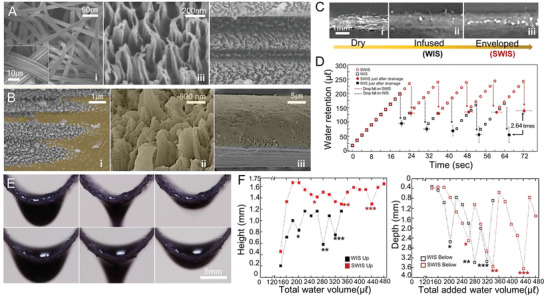
The water layers on the SWIS. SEM, cryo‐SEM, optical images before and after water wetting, and their characterization. A,i) A membrane of cellulose fibers 10–20 µm in diameter with ii) GNT structures and iii) two microchannels fully covered with GNTs fabricated by plasma etching. B) Cryo‐SEM images of i) water‐wet microchannels, ii) GNT structures, and iii) a single fiber enveloped by water. C) Optical side view images for i) dry and wet membranes of ii) WIS and iii) SWIS after dropping water of 60 µL on fabrics. D) A graph showing water retention volume in the SWIS and WIS membranes (0.5 × 2.3 cm^2^) with the water infusing time. Error bars indicate the standard deviation from three experiments. E) Sequential images of water height above the membrane just before, during and after a drop fall off the membrane on (top) the WIS and (bottom) the SWIS. F) Graphs for (left) the measured thickness of the water surface from the membrane center to the top and (right) to the bottom.

The cellulose membrane was a nonwoven fabric composed of microfibers with lobe‐shaped cross‐sections bonded together by mechanical entangling (**Figure** **2**A,i). The micro/nanostructures were evolved to the specific configurations through vacuum oxygen plasma etching on the fibrous membrane surface (Figure S1a, Supporting Information). With the initial 10 min of oxygen plasma processing, the nanopillar fibrils were formed on the fiber surface. The nanofibril became larger in length to increase the aspect ratio of the fibril due to the continuous bombardment of the high‐energy plasma ions. Then, they aggregated with neighboring nanofibrils to form a cluster that played a role of a self‐masking against oxygen plasma ion, resulting in the nanotrichome structures. The single nanofibril of the cluster also became a local mask to induce the nanogrooves along the nanotrichome surface. After 30 min of oxygen etching, GNT with a width of 207 ± 15 nm and a height of 659 ± 0.49 nm formed on the fiber surface (Figure 2A,ii and Figure S1b, Supporting Information). Note that not only the height of the GNT but also the pitch between the neighboring peaks were drastically increased (Figure S1c, Supporting Information). The oxygen plasma etched the cusp of the lobe on the cellulose fiber and developed microchannels along the fiber axial direction with 1.06 ± 0.14 µm deep and 1.79 ± 0.3 µm wide (Figure 2A,iii and Figure S1d, Supporting Information). Consequently, the nanofibrillation and its double self‐masking by oxygen plasma etching created the GNTs and microscale channels on the fiber surface. In addition, the mechanical strength maintained on the cellulose membrane with the GNT on which even though the intensity of the hydrophilic bonding groups were increased while the chemical composition was unaltered (Figure S1e,f, Supporting Information). Interestingly, the GNTs were morphologically similar to trichomes of pitcher plants which were reported to use water as an efficient lubricant (Figure S1g, Supporting Information).^[^ ^13^ ^]^ Protruding cell structures, short trichomes, covered the inner wall surface, which was suggested to support a stable, thick water layer on the pitcher plant such as *Heliamphora nutans* or *Sarracenia leucophylla*.^[^ ^14^ ^]^ As long as water was supplied by rain or humid air, the water lubricant surface maintained slippery even when scratched or pressured by footholds of prey insects and even tiny frogs.^[^ ^13^, ^15^ ^]^

When a GNT fiber was wet, water propagated first along the microchannels and then spread out through the GNT fibers (Figure 2B,i). The cryo‐SEM (scanning electron microscopy) images (Figure 2B,ii and Figure S2, Supporting Information) showed that individual GNT structures were wet first then the fiber surface got suffused with the thick water layer (Figure 2B,iii). The GNT microfiber appeared to have high surface energy to attract water molecules due to increased hydrophilicity contributed by the oxygen plasma etching and the newly added roughness by the GNT structure. Contrasted with the cellulose membrane without GNT, the rapid spreading of water droplet indicated the membrane with GNT fibers rendered superhydrophilic (Figure S3, Supporting Information). Water droplet spread primarily along the top surface on the GNT membrane, whereas it smeared into the fibrous network and delayed for surface spreading on the pristine membrane. When adding 60 µL of water to a dry membrane (i), water smeared between the fibrous web on the pristine membrane to form the WIS in which the fibers on the top surface area were still protruded through the water layer, resulting in partial wetting (ii). On the other hand, with the same volume of water infusion, the membrane with GNTs got fully infused over even the top surface to form the SWIS on which no fiber was pierced through the air‐water interface (Figure 2C and Movie S2, Supporting Information). The measurable water layer was seen with a thickness of ≈250 µm on the membrane with GNT, or SWIS membrane, while it was noticeably thinner on the water‐wet pristine membrane or WIS membrane. It was estimated that the SWIS membrane retained more water volume under a constant cycle of water adding and drainage (Figure 2D). The SWIS membrane kept considerable water volume (over 134 ± 0.4 µL per unit projected area of the membrane in mm^2^) which is up to 2.64 times larger than the WIS even after drainage. Notably, the drainage of both membranes was similar at 13.2 µL s^−1^ on the WIS and 12.7 µL s^−1^ on the SWIS. The substantial difference in water retention between the WIS and SWIS membranes could explained the hydrophilic GNT fibers on the top surface would hold a thick water layer (Figure 2C).^[^ ^6^ ^]^ The height of the water level was determined for both membranes bent with the initial curvature of 72 mm (Figure 2E). The SWIS membrane maintained a constant water level higher than the WIS which, however, the surface water level fluctuated remarkably before and after drainage. The average retained water height was 1.48 ± 0.12 mm on SWIS and 1.00 ± 0.24 mm on WIS (Figure 2F and Movie S3, Supporting Information). Take notice that the average length of the water suspended from the membrane just before the droplet falls off the membrane was similar (1.89 ± 1.1 mm), indicating that the GNTs did not affect the drain flux but improved water retention capability. The water affinity of the SWIS to retain more and thicker water would be estimated by the Wilhelmy balance method, which used experimentally determined vertical wetting angle and water‐air surface tension *γ*_water_ (Figure S4, Supporting Information).^[^ ^16^ ^]^ The wetting force *F*, equivalencing surface‐attracting force, for the meniscus along the vertical membrane was expressed as *F* = *γ*_water_ *l*cos *θ*_e_, where *l* is the contact length of the membrane and *θ*_e_ is equilibrium contact angle on a fibrous membrane. By substituting water surface tension *γ*_water_ (72 mN m^−1^) and the measured values of *θ*_e_, 37° ± 2.8° to the WIS and 6.1° ± 1.3° to the SWIS as well as the contact length (or a roughness factor) for both surfaces, *l*_GNT_ (2.65 ㆍ *l*_CF_) (Figure S5, Supporting Information), the wetting force for the SWIS (*F*_SWIS_ = 1.2 × 10^–1^ µN) was 3.3 times higher than that for the WIS (*F*_WIS_ = 0.36 × 10^–1^ µN). Thus, the modified surface topography of the membrane in the hierarchical dimension by GNT fibers allowed SWIS systems to have predominate water wetting power.^[^ ^17^ ^]^

### 2.2 Slippery, Water‐Lubricant Layer for Liquid Repellency

To repel immiscible liquids (i.e., oil), a membrane must be wetted preferentially with water, and the water layer must not be displaced or depleted by the oil. The stability of the water layer of membranes against oil was determined thermodynamically by the interfacial energy levels of the water surface (*E*_water_), oil surface (*E*_oil_), and water‐oil surface (*E*_water‐oil_). To ensure the water infusing layer, *E*_water_, and *E*_water‐oil_ should both be lower than *E*_oil_, or the energy difference of Δ*E*_1_ (=*E*_oil_ – *E*_water‐oil_) and Δ*E*_2_ (=*E*_oil_ –*E*_water_) must be positive, expressed as follows (see the details in Section SD1, Supporting Information).

(1) $$\Delta E_{1}\;=\;R\;\times \;\left(\gamma_{\mathrm{water}}\cos\theta_{\mathrm{water}}-\gamma_{\mathrm{oil}}\cos\theta_{\mathrm{oil}}\right)-\gamma_{\mathrm{water}-\mathrm{oil}}>0$$

(2) $$\Delta E_{2}\;=\;R\;\times \;\left(\gamma_{\mathrm{water}}\cos\theta_{\mathrm{water}}-\gamma_{\mathrm{oil}}\cos\theta_{\mathrm{oil}}\right)+\gamma_{\mathrm{oil}}-\gamma_{\mathrm{water}}>0$$

where *γ*_water_ and *γ*_oil_ are the surface tensions for the water and the oil, respectively. *γ*_water − oil_ is the interfacial tension at the water‐oil interface, *θ*_oil_ and *θ*_water_ are the equilibrium contact angle of oil and lubricating water on a flat solid surface, and *R* is the roughness factor. It was appraised that the water layer would not be stable against overlay oil on the flat cellulose sheet (*R* ≈ 1) as Δ*E_1_* and Δ*E_2_* were all negative values (Table SD1, Supporting Information), which was consistent with previous work.^[^ ^6^, ^18^ ^]^ On the fibrous membrane having WIS, the surface roughness increased with the microscale roughness, *R*_m_ = 1.34 ± 0.19, although both values were positive, Δ*E_2_* was still relatively small (≈6 mN m^−1^) for the highly viscous fuel oil (heavy fuel oil, HFO), of which the interfacial tension and the viscosity were ranged with ≈54–60 mN m^−1^ and 1000–10 000 cSt. On the other hand, on the GNT membrane having the hierarchical roughness in nano and microscale was estimated as *R*_mn_ = 4.0 ± 0.67, resulting in that Δ*E_1_* and Δ*E_2_* were all strongly positive greater than 160 mN m^−1^, referring that the GNT membrane would readily form the stable lubricating layer to support all immiscible liquids that were targeted.

**Figure 3:**
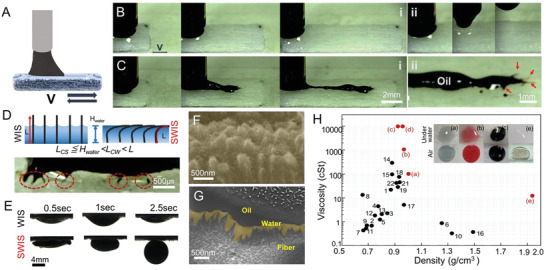
Water‐lubricating membrane for oil repelling in air and self‐cleaning underwater. A) A schematic showing the oil on the membranes under cyclic movement with speed (*v*). High‐speed camera images showing LSFO droplets lubricating on the WIS and the SWIS with three cycles with a constant speed after lowing the oil droplet to touch the surface. B) On the SWIS, no residue was seen after the third cycle and upon lifting the oil droplet. However, C) on top WIS membrane, the membrane was mobile (i), leaving the oil residue on the track due to the local pinning to the fibers discretely extruded (red arrow in (ii)). D) A schematic showing the water layer thickness (*H*_water_) versus the critical fiber thickness. *L*_CS_ and *L*_CW_ were the critical lengths for the fibers on the SWIS and the WIS, respectively. The bottom image shows the oily branches adhered to the fibers on the WIS (red circle). E) Optical images of oil droplets repelled on WIS and SWIS underwater. HFO was first dropped on both membranes in air and then immersed in water to explore the oil repellency behaviors. Cryo‐SEM images of F) a water‐wet GNT in the top view and G) LSFO on a water‐wet GNT in the cross‐sectioned view. Two schematics for each condition were inserted. H) A graph showing underwater repellency for immiscible liquids with a wide range of densities and viscosities listed in Table S1 in the Supporting Information. Liquids numbered (black dots) were investigated for underwater superoleophobicity (see references in the supplementary information), and selective liquids (red dots) were newly tested on the SWIS membrane as a) silicone oil 100 cSt, b) silicone oil 1000 cSt, c) LSFO, d) bunker C, and e) FC70, for which the underwater contact angle was more than 160°.

The cyclic friction behavior of oils further confirmed the stability of the SWIS (**Figure** **3**A). HFO was slid over without leaving any residue behind due to the low friction between the oil and the water surface (Figure 3B,i and Section SD2 and Movie S4, Supporting Information). The invariably maintaining water lubrication layer protected the SWIS against oil contamination even after three cycles of oil movement (Figure 3B,ii). Meanwhile, oil was pinned immediately upon moving over the WIS, and accumulated thick and wide as oil moved repeatedly (Figure 3C,i and Movie S4, Supporting Information). Microfibers on the WIS that did not submerge in water were discovered to be the lubrication‐discrete sites making oil residue branches (red arrowed, Figure 3C,ii). To achieve complete and robust repellency against highly viscous and adhesive oils, microfibers at the air‐water interface must not protrude above the lubricating water layer. The critical fiber length (*L*_CL_), above which the fiber submerged into water rather than piercing the air‐water interface, was calculated by considering the roughness on the membranes (Figure 3D, and see the details in Section SD 3, Supporting Information).^[^ ^19^ ^]^ *L*_CL_ for the SWIS and WIS membranes were 157 and 303 µm, respectively, meaning that the required water thickness (*H*_water_) on the SWIS to submerge fibers entirely was lower than on the WIS. With lower *L*_CL_, all fibers on SWIS were covered by the water layer, which was stable even with the certain pressure to keep the surface slippery against the heavy oil (Figures 1A and 3B). Due to higher *L*_CL_, the fibers on the WIS were not fully protected from being fouled by oil as oil stuck sites were revealed (red arrowed, Figure 3D). It was further explored that when the WIS membrane was contaminated with oil in air and immersed in water, the oil did not recede, firmly maintaining its contact line. However, the SWIS has immediately shoved away oil and it was completely detached before receding of the contact line within 0.5 s (Figure 3E and Figure S6, Supporting information). Cryo‐SEM analysis confirmed that the GNTs on a fiber (Figure 3F) were covered by water which in turn clearly separate the GNT structure and oil layer as a triple layer of GNT‐water‐oil was distinguished in the cross‐sectional image (Figure 3G). This was the direct evidence that the water layer strengthened by the GNT on a single fiber level contributed to the oil repellency via the stable water lubrication. The underwater repellency of the SWIS stayed perfect to various immiscible liquids, with a wide range of oil densities (0.65–1.98 g cm^−3^) and viscosities (0.45–10 000 cSt) (Figure 3H and Table S1, Supporting information). Note that no literature was dealt with the repellency against HFOs with extreme viscosity or higher density oils such as FC70 and low sulfur fuel oil (LSFO). The SWIS membrane was shown to repel the much viscous phase of the heavy oils in a form of irremovable goo as heavy oils were reported to lose their fluidic characteristics under the cold environment due to the rapid increase up to 100 times in viscosity as water temperature decreased to 5 from 25 °C.^[^ ^20^ ^]^

### 2.3 Collecting HFO by SWIS Scooper

**Figure 4:**
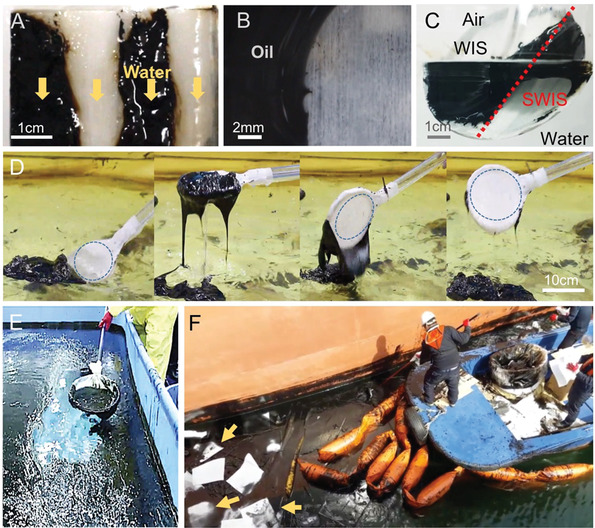
Oil repellency and recovery by the SWIS membrane. A) Self‐cleaning of HFO by water following the alternative pattern of WIS/SWIS/WIS/SWIS under air conditions. B) HFO residues after repeated dipping through the air–water interface on WIS and SWIS after 100 cycles at 17 °C. C) HFO oils on oil scooper patterned with WIS/SWIS showing that HFO was separated entirely only on the SWIS. Optical image of the oil scooping operation from D) a series of oil scooping‐releasing procedure (LSFO): oily residue with the thickness of ∼few mm (in black) was knocked off from the SWIS on which the surface was revealed clean (the blue circular area) E) a smaller reservoir (LSFO), and F) oil‐spilled site in seashore (HFO).

The strong oil repellency on the SWIS was demonstrated with the self‐cleaning behavior after being contaminated in air. By a single water flowing on the alternatively patterned surface, the oil on the SWIS region was removed, while the thick oil layer stuck on the WIS region (**Figure** **4**A and Figure S7a, Supporting Information). The oil was also not adhered to the SWIS region after 30 times cyclic dipping test at 37 °C and 100 times at 17 °C (Figure 4B and Figure S7b,c, Supporting Information). When the oil‐contaminated membrane with the patterned surface, assembled with a metal scoop frame (SWIS scooper), was immersed in water, the HFO detached quickly from only the SWIS region as it passed through the air‐water interface (Figure 4C and Movie S5, Supporting Information). The scooper with SWIS only was applied for the recovery of spilled HFO (LSFO) in water at 7 °C (Figure 4D and Movie S6, Supporting Information). When scooping the LSFO/water mixture together, the water drained out quickly through the membrane, leaving a thick LSFO mass on the SWIS scooper. The LSFO mass was removed from the scooper with a slight tilt, and the surface of the scooper returned to its original clean condition. Note that because thick LSFO below 20 °C exhibited non‐Newtonian fluid behavior,^[^ ^21^ ^]^ the collected LSFO detached from the SWIS scooper via a rolling‐off rather than sliding.^[^ ^22^ ^]^ After the test, the surface of the SWIS scooper remained clean.

In a pilot‐scale marine spill test, as the SWIS scooper was able to be used continuously without oil contamination, spilled oil was successfully collected at a recovery rate of more than 99% (Figure 4E and Figure S8 and Movie S7, Supporting Information). The SWIS scooper was also tested in a seashore HFO Bunker C fuel oil (BCFO) spill site (near the west coast of Mokpo city, Korea), proving the outstanding applicability in handling in‐field marine oil spills (Figure 4F). In comparison, oil absorbent sheets remained white (yellow arrows), indicating that HFO in a cold environment (average seawater temperature ≈11 °C, March 2017) could not be absorbed due to its viscosity. The collected volume of HFO with a single SWIS scooper in 10 min operation was similar to that removed by 900 absorbent sheets (3.6 m of stock height, Figure S9, Supporting Information). Therefore, the SWIS scooper could certainly replace a significant amount of absorbent sheets made of synthetic fiber, such as polypropylene and polyester. In addition, the collected HFO in the collection bottle could be reused since it does not have to be burned or buried, which substantially prevents secondary pollution.

## 3 Conclusions

Inspired by the trichome structure‐assisted water lubricating mechanism from natural plants, we fabricated a membrane with the slippery, water‐infused surface with grooved, nanotrichome through an oxygen plasma treatment. The GNT structures strengthened the surface wetting force to establish a thick water layer stably on the SWIS. The water lubrication on the SWIS membrane maintained the stability and robustness over immiscible liquids regardless of density and viscosity by forming the slippery, smooth water‐air interface. The robust and durable oil repellency performance of the SWIS membrane was demonstrated as the SWIS scooper by applying to collect the spilled oil in high recovery efficiency at laboratory and ocean sites.

The membrane system with the SWIS was easy to operate, inexpensive and scalable to manufacture (Figure S10, Supporting Information), and environmentally friendly. The SWIS membrane would be available in various applications, from simple home cleaning devices separating immiscible liquids from oily wastewater to motor‐powered oil skimming devices in larger‐scale operations.

## 4 Experimental Section

### Materials and Chemicals

A nonwoven fabric‐type membrane composed of cellulose fibers with a lobed cross‐section was purchased from Beak‐san Lintex, Korea. The fabric has a density of 45 g cm^−3^. Bunker C Fuel Oil (BCFO) and LSFO were provided by a petroleum company (Hyundai Oil Bank and GS Caltex, Korea). The densities were 0.98 and 0.94 g cm^−3^ and the pour points were both 10–20 °C.

### Plasma Etching of Cellulose Membrane

A reactive ion etching process was used to treat the membrane in custom‐made plasma equipment with a cathode 50 cm in diameter (J&L tech, Korea). The plasma voltage and power were set to 400 V and 224 W, at which plasma etching was performed with a flow rate of 100 sccm oxygen gas at 8 mTorr base pressure and 24 mTorr working pressure.

### Characterization of Water Behavior on the Membranes

The water layer thickness and water retention volume were measured by optical images taken in a side view of the membranes for the flat and curved configurations, respectively. Values on a flat membrane of 2.3 cm × 0.5 cm (Figure 2C,D) were calculated by measuring the volume of the falling drop below the membrane while infusing deionized water at 10 µL s^−1^ (recorded by the high‐speed camera). On a curved membrane of 2.5 cm × 0.5 cm (Figure 2E,F), the height and depth values were calculated by measuring the distances from the sample centerline to the top and bottom surfaces of water in the cross‐section, respectively. Every 40 µL of water‐soluble ink (black color)‐mixed deionized water was added to the membranes. Quantitative values were measured for the water wetting on the pristine and GNT cellulose membranes when the droplet reached equilibrium without moving. The captured images were quantitatively analyzed using ImageJ (National Institutes of Health).

### Oil Droplet Adhesion and Sliding Tests

Oil droplets were pressed on the membrane by lowering the syringe tip and then lifted gently. The sliding test was performed with LSFO droplets. As it dropped on the membrane surfaces, the membranes were moved for a distance of 15 mm at 5 mm s^−1^ for three cycles, during which surface oil fouling was observed by a high‐speed camera. The wetting angle of the immiscible liquids was measured underwater.

Surface analysis and SEM (Regulus 8230, Hitachi) was performed under an acceleration voltage of 2 kV on 45 s Pt‐coated cellulose membranes. RAMAN spectroscopy (Renishaw, In Via Raman Microscope) was performed for a 1 cm^2^ cellulose membrane and GNT membrane after wetting with 70 µL water. The intensities of the OH group of water at the top surface (depth = 0) and at a depth of 36 µm from the top were measured. Cryo‐SEM (Quanta 3D, FEI company) was performed under an acceleration voltage of 5 kV on cellulose membranes after freezing at −170 °C using liquified nitrogen.

### Oil Spill Removal Trial at the Pilot Scale and Marine Spill Simulation

A pilot‐scale test was performed with the SWIS‐covered scooper for the removal of 2000 L of LSFO in a cube‐shaped oil reservoir of 3 × 2 × 5 m^3^ (L × W × H). Scoopers of 30 cm in diameter and 5 cm in depth were manufactured for collecting the LSFO mess. The water surface temperature was maintained at 7 and 20 °C. Marine HFO (including BCFO) spill recovery was performed with the help of the Marine Pollution Response Team of the Korea Coast Guard near the west coast of Mokpo city in South Korea in March 2017.

## Conflict of Interest

The authors declare no conflict of interest.

## Supporting information

Supporting Information

Supplemental Movie 1

Supplemental Movie 2

Supplemental Movie 3

Supplemental Movie 4

Supplemental Movie 5

Supplemental Movie 6

Supplemental Movie 7

## Data Availability

The data that support the findings of this study are available in the supplementary material of this article.
