# Supplementary material for: Slippery, Water‐Infused Membrane with Grooved Nanotrichomes for Lubricating‐Induced Oil Repellency
Source: Adv Sci (Weinh). 2022 Feb 9;9(13):2103950. doi: 10.1002/advs.202103950 (PMC9069195; doi:10.1002/advs.202103950)
Supplement: Supplementary file 1 — Supporting Information [file ADVS-9-2103950-s005.pdf]

ADVANCED SCIENCE
Open Access

Supporting Information

for *Adv. Sci.,* DOI: 10.1002/advs.202103950

Slippery, Water-infused Membrane with Grooved Nanotrichomes for Lubricating-induced Oil Repellency

Young A Lee, Seohyun Cho, Seounkyun Choi, O-Chang Kwon, Sun Mi Yoon, Seong Jin Kim, Kyoo-Chul Park, Seok Chung and Myoung-Woon Moon**

Supporting Information

Slippery, Water-infused Membrane with Grooved Nanotrichomes for Lubricating-induced Oil Repellency

Young A Lee, Seohyun Cho, Seounkyun Choi, O-Chang Kwon, Sun Mi Yoon, Seong Jin Kim, Kyoo-Chul Park, Seok Chung & Myoung-Woon Moon**

This file includes:

Supporting discussion SD 1, SD 2, SD 3
Figure S1 to S10
Table S1
Captions for Movies S1 to S7
Supporting References (1 to 16)

Supporting Discussion

Discussion 1 (SD 1)

To form a stable water layer on a surface with roughness, the basic requirement is that the surface should be firmly wet by water (W) while repelling the oil (O), and two liquids should be immiscible as oil floats on water (WO). To determine whether a surface was wetted preferentially by water, we compared the total interfacial energy of the individual wetting configurations as described in the image modified from the previous work (Figure SD1).[1] Specifically, configuration $Conf_{oil}$ refers to the state where the textured surface was entirely wetted by oil. Configurations $Conf_{water}$ and $Conf_{water\text{-}oil}$ refer to the states where textured surfaces were entirely wetted by water with and without a fully wetted oil, respectively. By comparing the energy conditions in three configurations, $Conf_{water}$ and $Conf_{water\text{-}oil}$ were consistently lower than $Conf_{oil}$ in total energies.

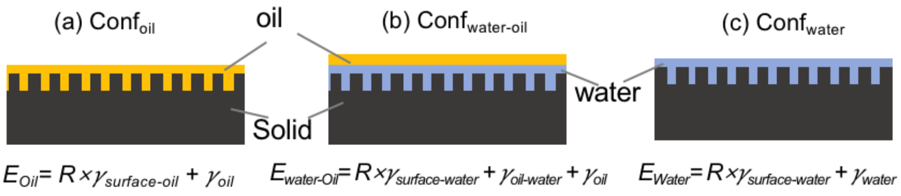

Figure SD1. Three configurations of water or oil on the textured surfaces; (a) oil only, and water (b) with and (c) without oil (or a targeting immiscible liquid) layer on water on the textured surfaces. The energy states were described for each configuration.

In the above configuration (Fig. SD1), the interfacial energy levels of the water surface (E_{Water}), oil surface (E_{oil}), and oil-water surface ($E_{water\text{-}oil}$) represent the total interfacial energies per unit area of each configuration. γ_{water} and γ_{oil} are the surface tensions for the water and the oil on the solid surface, respectively, $\gamma_{water-oil}$ is the interfacial tension at the oil-water interface, and R represents the roughness *(the ratio between the actual and projected surface areas of the textured solids)*.To derive a working condition of this energy relationship, the following assumptions were made:

1) The fluid layer covers the surface features (i.e., H > h);
2) The thickness of the fluid layer is much less than the capillary length of the fluid;
3) The surface roughness is uniformly distributed for each configuration;
4) Oil (targeting immiscible liquid) and water are chemically nonreactive with the solid (cellulose).

The total energy for each configuration can be expressed as

$$E_{water\text{-}oil} = R \times \gamma_{surface-water} + \gamma_{water-oil} + \gamma_{oil} \quad (s1)$$

$$E_{water} = R \times \gamma_{surface-water} + \gamma_{water} \quad (s2)$$

$$E_{oil} = R \times \gamma_{surface-oil} + \gamma_{oil} \quad (s3)$$

To obtain the stable condition for $\mathrm{Conf}_{water\text{-}oil}$, with (s3) and (s1), we have $\Delta E_1 = E_{oil} - E_{water\text{-}oil} > 0$, which can be further expressed with the use of the Young equation, as such,

$$\Delta E_1 = R \times (\gamma_{water} \cos\theta_{water} - \gamma_{oil} \cos\theta_{oil}) - \gamma_{oil-water} > 0 \quad (s4)$$

where θ_{oil} and θ_{water} are the equilibrium contact angles of oil and water on a flat solid surface, respectively.

Similarly, to find the conditions such that Conf_{water} is always stable over Conf_{oil}, with (s3) and (s2) we have $\Delta E_2 = E_{oil} - E_{water} > 0$, which can be further expressed as

$$\Delta E_2 = R \times (\gamma_{water} \cos\theta_{water} - \gamma_{oil} \cos\theta_{oil}) + \gamma_{oil} - \gamma_{water} > 0 \quad \text{(s5)}$$

Satisfying both (s4) and (s5) ensures stable lubricating water layer formation on the surface. In contrast, when neither (s4) nor (s5) are satisfied, water is displaced by oil. To verify these design criteria, we explored a number of solid/water/oil type combinations and compared these results with the governing relationships as above. (s4) and (s5) were estimated based on measured values and the reference values (see Table SD1).

1. The static contact angle of liquids on a flat surface

Static contact angles were measured on cellulose sheets with nanoscale roughness of a few nm, as shown in Figure SD2.

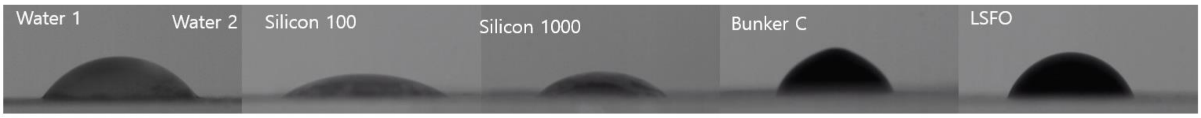

Figure SD2. Optical images of the static contact angles for water and oils on the cellulose sheet. (having a roughness of ~2.5 nm).

2. Roughness factor, *R*, on the solid configurations as shown in Figure SD3

1) Flat: *R*=1. The flat substrate was prepared with a cellulose sheet composed of nanocrystalline cellulose crystals, as shown in the AFM images with an average roughness of 2.5 nm.
2) The roughness by microfibers: R_m=1.501±0.104. The roughness was measured by the measured value of fiber geometries and spacing between the fibers.
3) The hierarchical roughness by nanoscale roughness nested on the individual fibers on the top surface of the membrane (see also the right in Fig. SD3). R_{mn} = 4.043±0.669

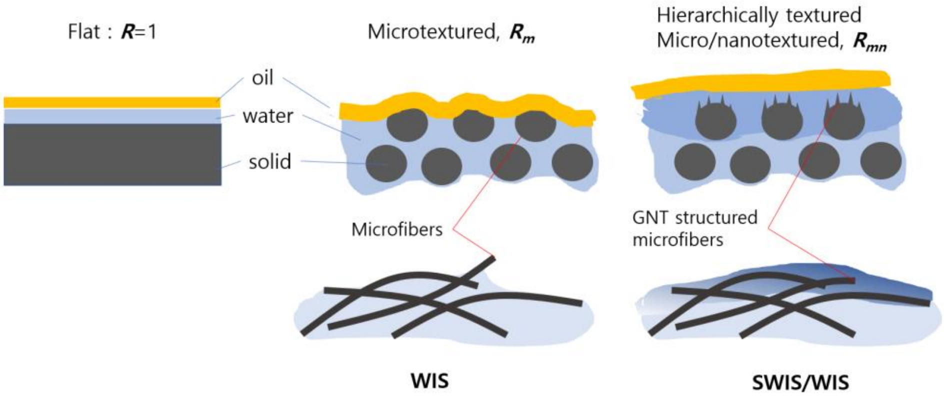

Figure SD3. Solid configurations with correspondent roughness factors: flat, microtextured by microfibers, and hierarchical (micro/nanotextured) configurations.

It was shown that these relationships do not agree favorably with the conditions on the flat solid, as ΔE_1 and ΔE_2 are all negative values, which agrees well with the result shown in the system that oil floated on the water-covered Si with R=1(5). On the microtextured surface of the fibrous cellulose membrane, those values became positive but still lower in the energy difference for the LSFO and Bunker oil due to the high interfacial tension. On the GNT surface, ΔE_1 and ΔE_2 were strongly positive, indicating that the water layer was always stable with or without an oil layer on the SWIS.

Table SD1. Comparison of the experimental estimation for the total energies for three configurations with solid roughness and five different oils with high viscosity. The parameters were measured at ambient temperature (23-25 °C). The dimensional unit for the surface tensions and energies is mN/m, and the contact angle is degrees (°). The surface and interfacial tensions for LSFO and bunker C were measured by the pendant drop method at ambient temperature.

Solid	Lubricant	Liquid(oil)	γ_{water}	γ_{oil}	$\gamma_{water-oil}$	θ_A	θ_B	ΔE_1	ΔE_2
Flat	Water		72			38.3			
		Silicon100	72	21	43	38.3	28.8	-4.9	-12.9
		Silicon1000	72	21	43	38.3	40.0	-2.6	-10.6
		LSFO	72	23	54	38.3	34.7	-16.4	-11.4
		BUNKER C	72	27	60	38.3	42.5	-23.4	-8.4
		FC 70	72	18	56	38.3	17.8	-16.7	-11.6
Micro	water		72			36.7			
		Silicon100	72	21	43	36.7	28.8	23.9	1.7
		Silicon1000	72	21	43	36.7	40.0	27.8	4.8
		LSFO	72	23	54	36.7	34.7	12.0	3.0
		BUNKER C	72	27	60	36.7	42.5	4.3	5.7
		FC 70	72	18	56	38.3	17.8	13.0	-1.2
Micro +Nano	water		72			6.1			
		Silicon100	72	21	43	6.1	28.8	169.7	161.8
		Silicon1000	72	21	43	6.1	40.0	179.0	171.0
		LSFO	72	23	54	6.1	34.7	156.7	161.7
		BUNKER C	72	27	60	6.1	42.5	146.7	161.7
		FC 70	72	18	56	6.1	17.8	161.8	163.8

Discussion 2 (SD 2)

Details of heavy oils

SWISs with GNT fibers presented a unique response to thick heavy fuel oil (HFO) composed of nonhomogeneous mixtures of carbon, sulfur, nitrogen, metals, and waxes, which are strongly adhesive and highly viscous.[2] Here, two types of HFO were chosen with sulfur content with high density (~0.98 g/cm^3): high sulfur oil of Bunker C fuel oil (BCFO, sulfur~3.5%) and low sulfur fuel oil (LSFO, sulfur < 0.5%). Furthermore, the LSFO is known for the new type of marine fuel oil adapted from 2020 by the International Maritime Organization (IMO). July on 2020 in Mauritius coast, LSFO was leaked from bulk carrier Wakashio, but a recovery solution was not found, especially in cold environments, due to the high pouring temperature and high content of waxy components in the oil.[3]

Discussion 3 (SD 3)

To prevent the oil from contacting the fibers during lubrication of oil over the membrane, the water lubricating layer should be thick enough to support the oil pressure. It was observed that the fibers that appeared on the top surface were covered by water as the water was added continuously, suggesting that the thickness of the water layer would be thicker than the length of the fibers that were straight or bent on the top surface, and the oil residue may not foul the lubricating surface. The fibers or rods remained immersed below the water surface as they buckled by the capillary force. Buckling occurs when the capillary force, $2\pi r\gamma \cdot f \cdot cos\theta$, exceeds the critical load (Figure SD4), $(\pi/2)^2 \cdot EI_F/L_F^2$, where L_F and $EI_F \sim E\, r^4$ are the length and the bending stiffness of the microfiber clamped on one end, respectively, and r is the radius of the fiber. It was suggested that the fiber buckles if it is longer than a critical length[4] :

$$L_{crit} = \frac{\pi}{4\sqrt{2}} \sqrt{\frac{E \cdot r^3}{\gamma \cdot f \cdot cos\theta}}$$

If $L_F < L_{crit}$, the fiber stays straight and pierces the air-water surface, whereas if $L_F > L_{crit,}$ the fiber buckles to a deflected state underwater. Therefore, the water layer thickness satisfies the condition $H_{water} > L_{crit}$ ($H_{water} > L_{crit}$ for $L_F > L_{crit}$ (buckled) and $H_{water} > L_F$ for $L_F < L_{crit}$ (straight)) (mid, Fig. SD3), and water always covers the fibers with no piercing. In our system, Young's modulus of the cellulose fibers, E = 23 GP[5], the radius of fibers is approximately 10 mm, and the water surface tension is γ=72 mN/m^2. With these parameters, the critical length for cellulose fiber on WIS (L_{CW}) is approximately 303 μm, indicating that the water height needs to reach over 303 μm for complete coverage of the WIS membrane to avoid contact between the oil and pierced WIS fibers (Fig. 3A from the main text). On the other hand, the fibers on the SWIS membrane can be considered in the wetting state of the Wenzel condition with nanoscale roughness on the GNT fiber. Then, the critical length for a

GNT fiber on SWIS being buckled (L_{CS}) is~157 μm and lower than the water layer thickness measured on the SWIS membrane (~250 μm) (Fig. 2C), indicating that with even 157 μm of the water height, water can cover the entire fiber regardless of fiber geometries on the SWIS.

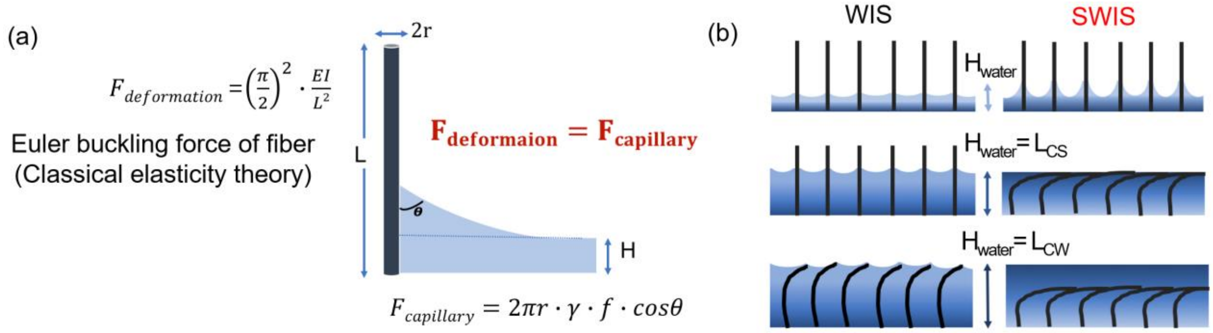

Figure SD4. (a) A schematic showing the force balance on a single fiber by water. The Euler buckling force for the fiber, $F_{deformation}$, is equivalent to the force induced by the water capillarity, $F_{capillary}$. (b) The buckling condition for the fibers on the surface conditions. As increases the height of the water, H_{water}, the WIS (left) having the microtextured fibers needs the critical length of L_{CW}, which is higher than that (L_{CS}) for the SWIS (right) having the GNT on the microtextured fibers.

Supporting Figures

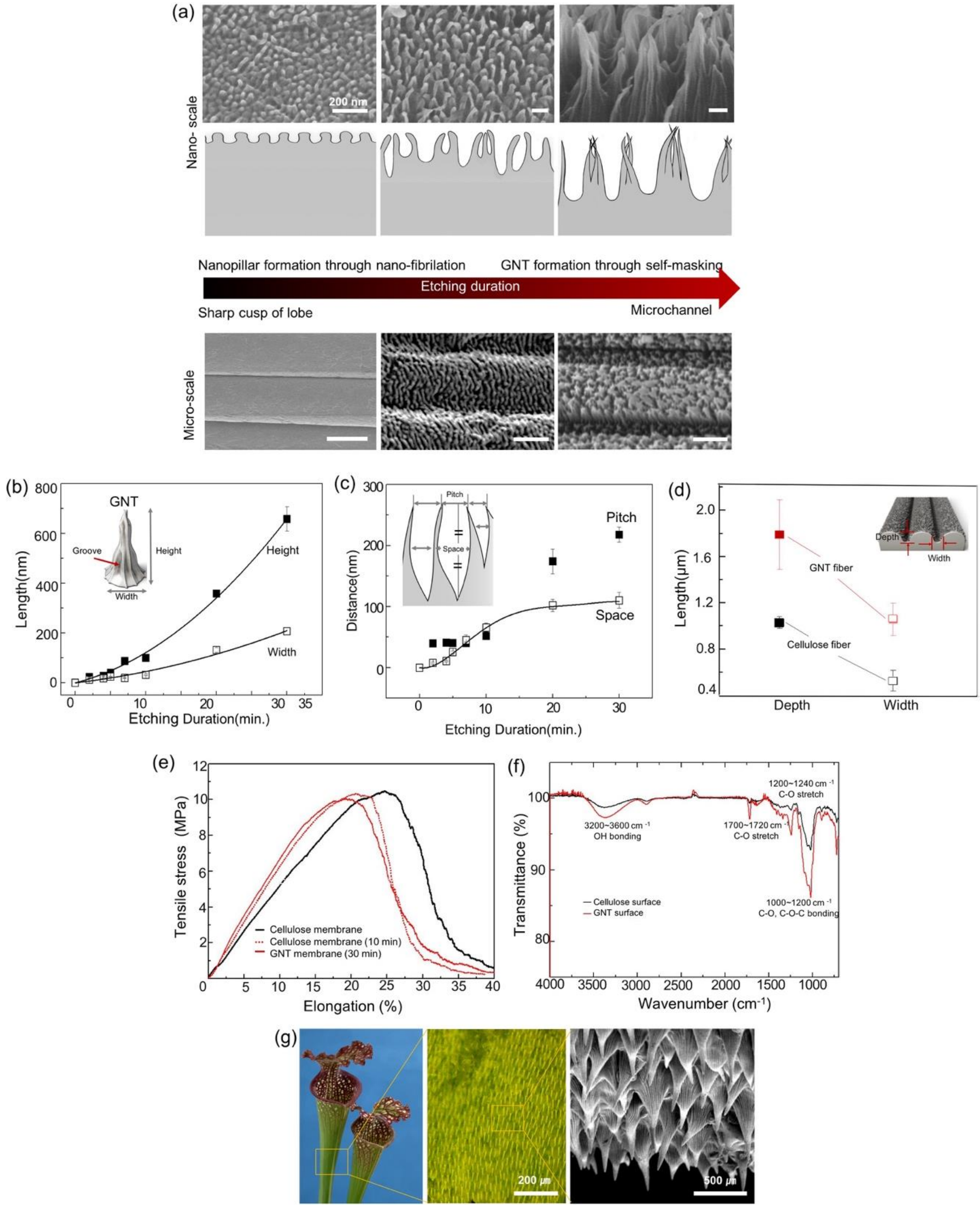

Figure S1. The GNT structural formation mechanism. (a) SEM images and schematics explaining the detailed formation mechanism through oxygen plasma treatment duration. Variations in (b) the height and the width of nanostructures and (c) the pitch and the inter-fibril space between the neighboring nanostructures with etching duration. Error bars indicate the standard deviation (d) Variations in the depth and width of the microchannels. Error bars indicate the standard deviation. (e) The measured graph for the 3 different plasma exposures of pristine and plasma duration of 10 and 30 min under tensile loading according to ISO

13934[6]. (f) The FT-IR spectra measured on the cellulose membrane and GNT membrane (30 min treated and aged for more than 12 months). After the GNT formation by the oxygen plasma treatment for 30 min, the apparent increase in the intrinsic chemical bonding groups was detected in several specific bonding sites of O-H, C-O, and C-O-C while no noticeable new bonding was detected. (g) The pitcher plant, *Sarracenia leucophylla,* and inner trichomes. Error bars indicate the standard deviation from 3 experiments.

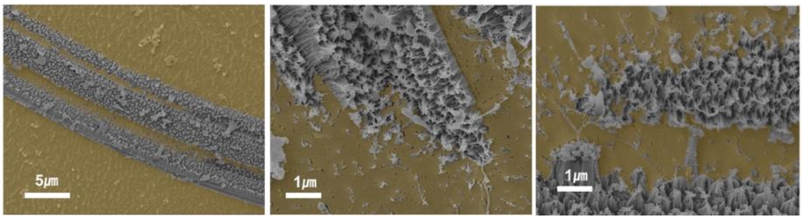

Figure S2. Cryo-SEM images showing GNT fibers of SWIS membrane wetting of both microchannels and GNTs. False color in yellow indicated water.

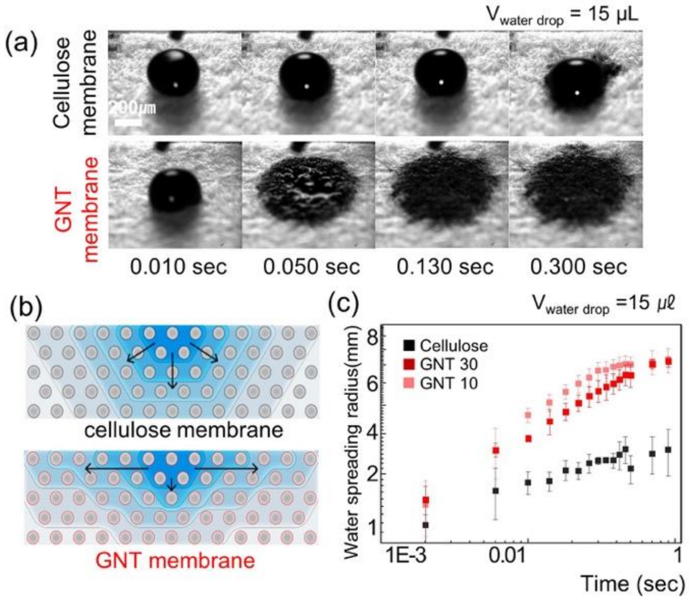

Figure S3. (a) High-speed camera images of deionized water droplets spreading on the untreated membrane (WIS) and the plasma-treated membrane (SWIS). The water droplet on the cellulose membrane did not spread and maintained the initial water contact angle until 0.130 seconds at which completely spread on the SWIS membrane, indicating that the GNT membrane became superhydrophilic. (b) Cross-sectional schematic illustrations of horizontal and vertical wicking (c) A graph for water drop spreading radius (WDR). The diameter was approximated at the two most distant points passing through the drop center. Error bars indicate the standard deviation from 3 experiments.

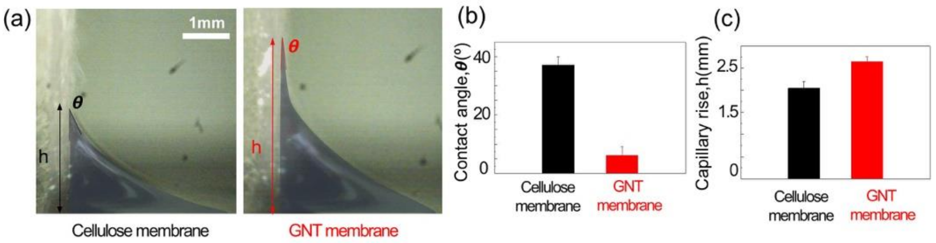

Figure S4. (a) Vertical capillary rising of deionized water on the WIS membrane and SWIS membranes. Graphs showing (b) the water contact angle and (c) the final height of capillary rise. Error bars indicate the standard deviation from 3 experiments.

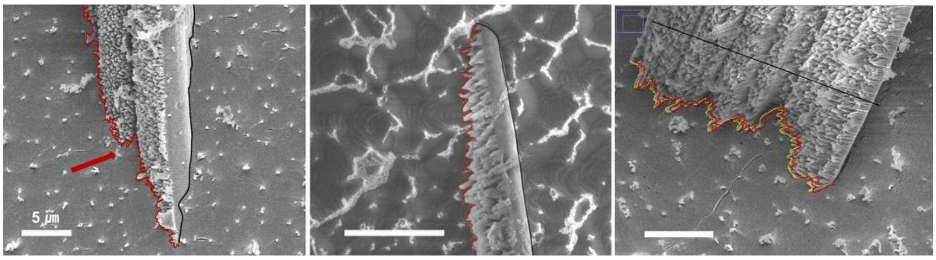

Figure S5. Cryo-SEM images of three fibers with and without GNT, which showed the increase in perimeter on the GNT side. The average length ratio defined as the perimeter on the GNT side (l_{GNT} along the red line) / that on the pristine side (l_{CF} along the black line) was l_{GNT} / l_{CF} =2.65.

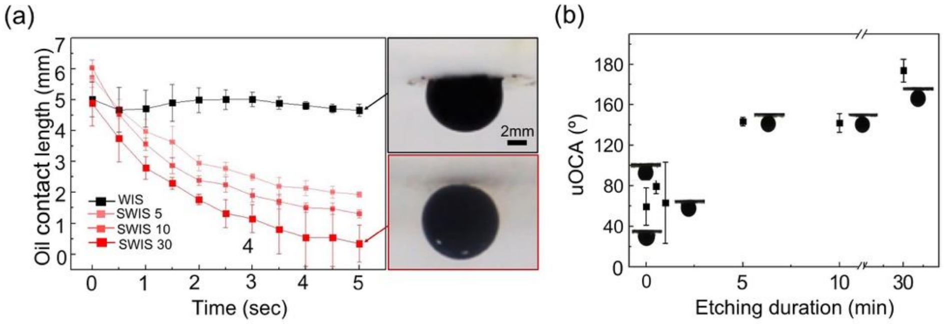

Figure S6. (a) Changes in the oil contact line of BCFO droplet and images (right) on both the WIS (upper) and the SWIS (lower) with the different plasma duration (SWIS 30 is a 30 min-plasma-treated SWIS). (b) The underwater oil contact angle (uOCA) with plasma treatment. After contaminated by BCFO in air, membranes were immersed into the water. Error bars indicate the standard deviation from 3 experiments.

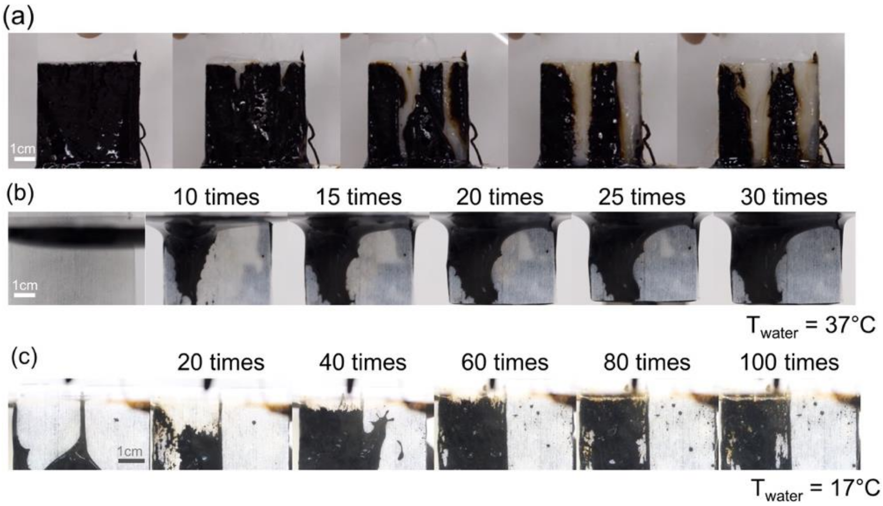

Figure S7. Separation of the BCFO mess off the patterned WIS/SWIS. (a) Washing by pouring water on the patterned membrane (WIS-SWIS-WIS-SWIS). Durability test of repeated dipping of the patterned fabric (WIS/SWIS) with BCFO mess after (b) 30 dips in T_W= 37 °C water and (c) 100 dips in 17 °C water.

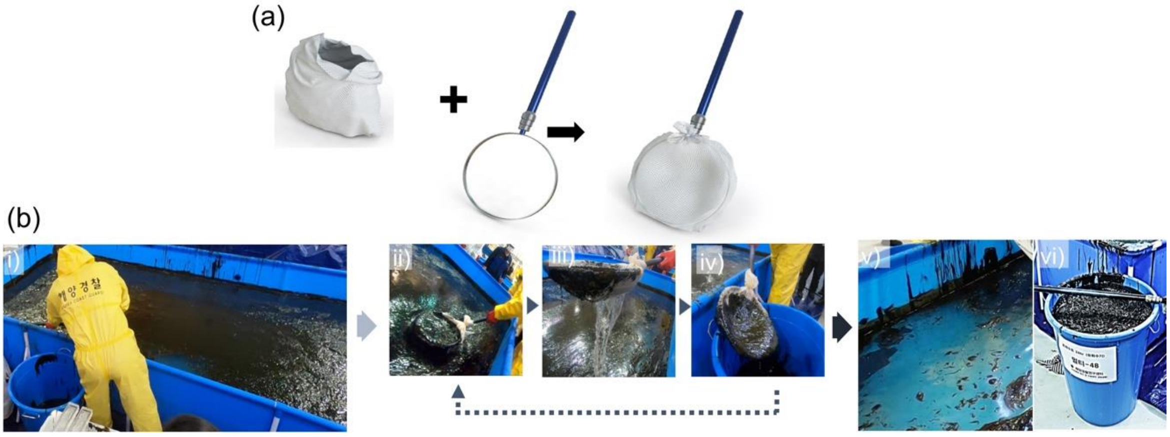

Figure S8. LSFO collection with SWIS: (a) A schematic of the SWIS scooper in (b) pilot-scale LSFO cleaning trial using the NFC scooper in cold water T_{water}= 7° from (i) LSFO spilled reservoir; (ii) scooped water and LSFO mixture, (iii) draining of water, (iv) pouring of collected LSFO into a collecting bucket, (v) continuously reuse of the clean scooper, and (vi) the collecting bucket full of collected LSFO.

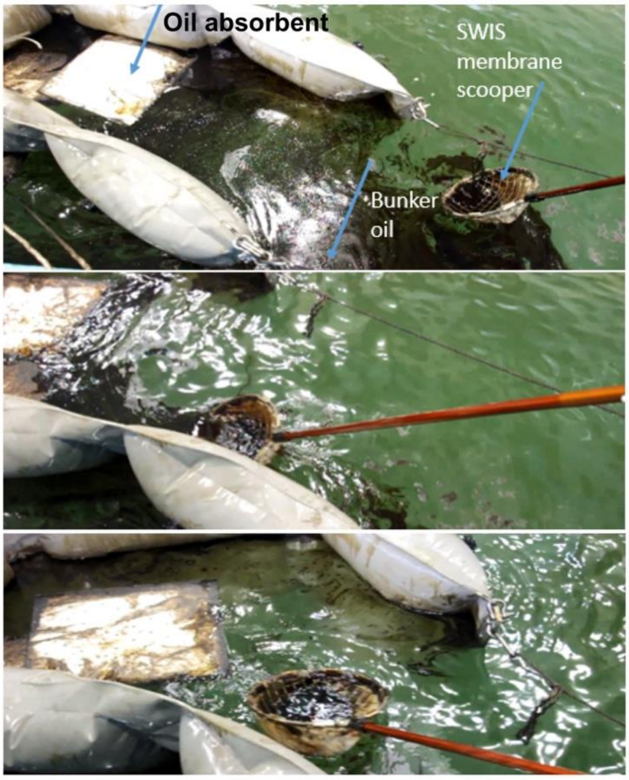

Figure S9. The comparison of the oil collecting efficiency between an oil absorbent (3M) and the SWIS membrane scooper for bunker B fuel oil shows that the oil volume collected by the single SWIS membrane-based scooper after 10 min of operation was estimated to be similar to that absorbed by 900 sheets of oil absorbents or 3.6 meters in stock height.

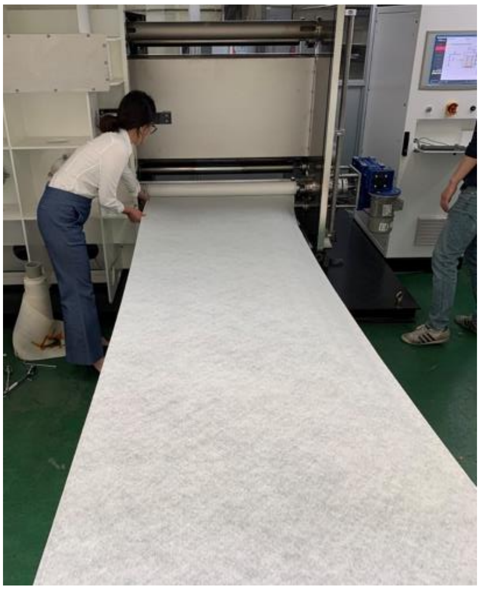

Figure S10. Mass producible roll-to-roll plasma equipment with GNT cellulose fabrics of 1 m in width and 100 m in length.

Table S1. References used for **Figure 3H**

No.		Oil	Supporting references
1	●	Crude	[7]
2	●	Gasoline	[7a,b,c, 8]
3	●	Diesel	[7b,c,d, 8a, 9]
4	●	Hexadecane	[8a, 10]
5	●	Cyclohexane	[8b, 11]
6	●	1,2-dichloroethane	[7b, 8a, 10d, 12]
7	●	Hexane	[7b,c,d,f, 9, 10d,e]
8	●	Petroleum ether	[7b,c,d, 8a, 9, 12d, 13]
9	●	Isooctane	[7f, 9]
10	●	Dichloromethane	[7f, 8b, 10e, 14]
11	●	Heptane	[13]
12	●	Dodecane	[12e]
13	●	Kerosene	[7f, 8b, 12e]
14	●	Motor oil	[7a]
15	●	LVO 100	[10d, 12d]
16	●	Chloroform	[8b, 12a,e]
17	●	Methylpolysiloxane(silicon)	[7g, 15]
18	●	Soybean	[8b, 9, 12e, 16]
19	●	Corn oil	[7a]
20	●	peanut oil	[10e]
21	●	Olive oil	[7f,g]
(a)	●	Silicon 100	-
(b)	●	silcon1000	-
(c)	●	Bunker C	-
(d)	●	LSFO	-
(e)	●	FC70	-

Supporting Movies

Movie S1. Dynamic response of oil droplets on the WIS (left) and the SWIS (right) as the droplets are lowered onto the surfaces and lifted

Movie S2. Water retention and drainage on the WIS (left) and the SWIS (right) membranes under continuous water addition and drainage

Movie S3. Water retention and drainage on the curved WIS (left) and SWIS (right) membranes under continuous water addition and draining (radius of curvature =72 mm)

Movie S4. Cyclic sliding test of oil droplets on the WIS (top) and the SWIS (bottom) membranes

Movie S5. Removal of BFCO on the SWIS scooper with alternatively patterned GNT structures

Movie S6. Oil collection procedure of LSFO from the SWIS membrane, showing a knock-off mechanism

Movie S7. Continuous operation of oil scooping by the SWIS scooper in large-scale oil reservoirs

Supporting References

[1] T-S Wong, S. H. Kang, S. K. Y. Tang, E. J. Smythe, B. D. Hatton, A. Grinthal, J. Aizenberg, *Nature* **2011**, 477, 7365

[2] M. Fingers, *Oil spill Science and Technology,* GPP, Houston, USA **2010**

[3] a) Denis Lweis, How Mauritius is cleaning up after major oil spill in biodiversity hotspot, https://www.nature.com/articles/d41586-020-02446-7, August, **2020** ; b) K. R. Sørheim, P. S. Daling, D. Cooper, I. Bust, L. G. Faksness, D. Altin, T.-A. Pettersen, O. M. Bakken, *Characterization of Low Sulfur Fuel Oils (LSFO) – A New Generation of Marine Fuel Oils-OC2020 A-050,* SINTEF, Norway **2020** ; c) T.-A. Patterson, K. R. Sørheim, M. Johnsen, Physico-chemical characterization and weathering properties of IM-5 Wakashio - OC2021 A-034, SINTEF, Norway **2021**

[4] a) S. Neukirch, B. Roman, B.D. Gaudemaris, J. Bico, *Mech. Phys. Solids* **2007**, 55 ; b) Z. Sheng, H. Wang, Y. Tang, M. Wang, L. Huang, L. Min, H. Meng, S. Chen, L. Jiang, X. Hou, *Sci. Adv.* **2018**, 4, eaao6724

[5] Sandip Basu, Tensile deformation of fibers used in textile industry : *Agilent Technologies Application Note*, 5991-0274EN **2012**

[6] International Standard, Textiles - Tensile properties of fabrics - Part 1: Determination of maximum force and elongation at maximum force using the strip method, ISO 13934-1: 1999(E)

[7] a) F. E. Ahmed, B. S. Lalia, N. Hilal, R. Hashaikeh, Desalination **2014**, 344b) Z. Xue, A. Wang, L. Lin, L. Chen, M. Liu, L. Feng, L, Jiang, Adv. Mater. **2011**, 23 ; c) A. Gao, L.-P. Xu, Z. Xue, L. Feng, J. Peng, Y.Wen, S. Wang, X. Zhang, Adv. Mater. **2014**, 26 ; d) N. Liu, Y. Chen, F. Lu, Y. Cao, Z. Xue, K. Li, L. Feng, Y. Wei, ChemPhysChem **2013**, 14 ; e) H.-C. Yang, Y. Xie, H. Chan, B. Narayanan, L. Chen, R. Z. Waldman, S. K. R. S. Sankaranarayanan, J. W. Elam, S.B . Darling, ACS Nano **2018**, 12, 8 ; f) S. Yuan, C. Chen, A. Raza, R. Song, T.-J. Zhang, S. O. Pehkonen, B. Liang, Chemical Engineering Journal **2017**, 328 ; g) Y. Cai, Q. Lu, X. Guo, S. Wang, J. Qiao, L. Jiang, Adv. Mater. **2015**, 27 ; h) S. Gao, P. Liu, F. Zhang, W, Zhang, S. Yuan, J. Li, J, Jin, Adv. Mater. **2016**, 28

[8] a) P.-C. Chen, Z.K. Xu, Scientific Reports **2013**, 3, 2776 ; b) C. Zhou, J. Chen, K. Hou, A. Zao, P. Pi, X. Wen, S. Xu, Chemical Engineering Journal **2016**, 301 ; c) L. Zhang, Z. Zhang, P. Wang, NPG Asia Materials **2012**, 4, e8

[9] F. Zhang, W. B. Zhang, Z. Shi, D. Wang, J. Jin, L. Jiang, Adv. Mater. **2013**, 25, 30

[10] a) A. K. Kota, G. Kwon, W. Choi, M. Mabry, A. Tuteja, *Nature Communications* **2012**, 3, 1025 ; b) J. Li, Q. H. Qin, A. Shah, R. H. A. Ras, X. Tian, V. Jokinen, Sci. Adv. **2016**, 2, e1600148 ; c) P. S. Brown, O. D. L. A. Atkinson, J. P. S. Badyal, ACS Appl. Mater. Interfaces **2014**, 6, 10 ; d) L. Zhang, Y. Zhong, D. Cha, P. Wang, Scientific Reports **2013**, 3, 2326 ; e) J. Song, L. Huang, Y. Lu, X. Liu, X. Deng, X. Yang, S. Huang, J. Sun, Z. Jing, I. P. Parkin, Scientific Reports **2016**, 6, 31818 ; f) Q. Liu, A. A. Patel, L. Liu, ACS Appl. Mater. Interfaces **2014**, 6,12 ; g) H. Yoon, S.-H. Na, J.-Y. Choi, S. S. Latthe, M. T. Swihart, S. S. Al-Deyab, S. S. Yoon, Langmuir **2014**, 30, 39

[11] Y. Liu, L. Zhao, J. Lin, S. Yang, Sci. Adv. **2019**, 5, eaax0380

[12] a) J. Yong, F. Chen, Q. Yang, D. Zhang, U. Farooq, G. Du, X. Hou, J, Mater, Chem. A, **2014**, 2 ; b) X. Meng, M. wang, L. Heng, L. Jiang, Adv. Mater. **2018**, 30 ; c) L.-P. Xu, J. Zhao, B. Su, X. Liu, J. Peng, Y. Liu, H. Liu, G. Yang, L. Jiang, Y. Wen, X. Zhang, S. Wang, Adv. Mater. **2013**, 25 ; d) T. Du, S. Ma, X. Pei, S. Wang, F. Zhou, Small **2017**, 13 ; e) Z. Lian, J. Xu, Z. Wang, Z. Yu, Z. Weng, H. Yu, Langmuir **2018**, 34,9

[13] B. Xue, L. Gao, Y. Hou, Z. Liu, L. Jiang, Adv. Mater. **2013**, 25

[14] X. Tang, Y. Si, J. Ge, B. Ding, L. Liu, G. Zheng, W. Luo, J. Yu, Nanoscale **2013**, 5

[15] Y. Huang, M. Liu, J, Wang, J, Zhou, L. Wang, Y. Song, L. Jiang, Adv. Funct. Mater. **2011**, 21

[16] A. Raza. B. Ding, G. Zainab, M. El-Newehy, S. S. Al-deyab, J, Yu, J. mater. Chem. A, **2014**, 2
